# A high content, phenotypic ‘scar-in-a-jar’ assay for rapid quantification of collagen fibrillogenesis using disease-derived pulmonary fibroblasts

**DOI:** 10.1186/s42490-019-0014-z

**Published:** 2019-06-28

**Authors:** Robert B. Good, Jessica D. Eley, Elaine Gower, Genevieve Butt, Andrew D. Blanchard, Andrew J. Fisher, Carmel B. Nanthakumar

**Affiliations:** 1 Fibrosis Discovery Performance Unit, Respiratory Therapy Area, Medicines Research Centre, GlaxoSmithKline R&D, Gunnels Wood Road, Stevenage, SG1 2NY UK; 2 Institute of Transplantation, Newcastle upon Tyne Hospitals NHS Trust and Institute of Cellular Medicine, Faculty of Medical Sciences, Newcastle University, Newcastle, UK

**Keywords:** Scar-in-a-jar, Extracellular matrix, ECM, Fibrosis, Fibroblast, Pulmonary fibrosis, Idiopathic pulmonary fibrosis, IPF, Phenotypic screening, High content imaging, Drug discovery, Assay development, Collagen type I, Collagen

## Abstract

**Background:**

Excessive extracellular matrix (ECM) deposition is a hallmark feature in fibrosis and tissue remodelling diseases. Typically, mesenchymal cells will produce collagens under standard 2D cell culture conditions, however these do not assemble into fibrils. Existing assays for measuring ECM production are often low throughput and not disease relevant. Here we describe a robust, high content, pseudo-3D phenotypic assay to quantify mature fibrillar collagen deposition which is both physiologically relevant and amenable to high throughput compound screening. Using pulmonary fibroblasts derived from patients with idiopathic pulmonary fibrosis (IPF), we developed the ‘scar-in-a-jar’ assay into a medium-throughput phenotypic assay to robustly quantify collagen type I deposition and other extracellular matrix (ECM) proteins over 72 h.

**Results:**

This assay utilises macromolecular crowding to induce an excluded volume effect and enhance enzyme activity, which in combination with TGF-β_1_ stimulation significantly accelerates ECM production. Collagen type I is upregulated approximately 5-fold with a negligible effect on cell number. We demonstrate the robustness of the assay achieving a Z prime of approximately 0.5, and % coefficient of variance (CV) of < 5 for the assay controls SB-525334 (ALK5 inhibitor) and CZ415 (mTOR inhibitor). This assay has been used to confirm the potency of a number of potential anti-fibrotic agents. Active compounds from the ‘scar-in-a-jar’ assay can be further validated for other markers of ECM deposition and fibroblast activation such as collagen type IV and α-smooth muscle actin exhibiting a 4-fold and 3-fold assay window respectively.

**Conclusion:**

In conclusion, we have developed ‘scar -in-a-jar is’ into a robust disease-relevant medium-throughput in vitro assay to accurately quantify ECM deposition. This assay may enable iterative compound profiling for IPF and other fibroproliferative and tissue remodelling diseases.

## Background

Tissue remodelling characterised by dysregulated extracellular matrix (ECM) deposition is a hallmark feature of numerous human pathologies including cancer and fibroproliferative diseases such as pulmonary fibrosis, chronic kidney disease (CKD) and non-alcoholic steatohepatitis (NASH) [1, 2]. In the lung, idiopathic pulmonary fibrosis (IPF) presents as a chronic, progressive condition and is the most common form of interstitial lung disease (ILD) [1]. IPF is characterised radiologically by the presence of honeycomb lung on high-resolution computed tomography (HRCT) and histologically by the appearance of fibroblastic foci [1] containing myofibroblasts in a collagen-dense ECM. Ultimately, these structural changes in pulmonary tissue architecture result in extensive remodelling of the lung parenchyma, leading to loss of lung function and causing death due to respiratory failure [3]. Diagnosis rates of IPF are expected to increase following the approvals of two therapies, Nintedanib and Pirfenidone, but only a subset of patients qualify for treatment based on lung function criteria and both drugs exhibit significant side effects [4]. With a prevalence rate of 50 in 100,000 in the UK [5] and an average life expectancy estimated to be less than 3 years [6], there is an urgent need to develop new medicines with improved efficacy and tolerability profiles for patients with pulmonary fibrosis [5, 6].

Fibrosis is characterised by the accumulation of myofibroblasts in ECM rich lesions. These myofibroblasts largely originate from resident tissue fibroblasts [7], express high levels of α-smooth-muscle actin (SMA), and contribute to fibrogenesis through the elevated production of ECM proteins including collagens and fibronectin [8]. One of the most well studied growth factors responsible for mediating the activation of resident fibroblasts is the pleiotropic cytokine, transforming growth factor (TGF)-β_1_. Known to possess chemotactic, proliferative and pro-fibrotic properties, TGF-β_1_ is produced by a number of cell types within the lung, such as neutrophils, alveolar macrophages, epithelial cells, endothelial cells and fibroblasts [9]. A TGF-β_1_-induced collagen type I deposition assay termed ‘scar-in-a-jar’ has been previously described [10, 11] enabling the acceleration of mature ECM deposition in vitro. Unlike previous assays which have used soluble collagen production as a surrogate marker of ECM production, or chronic TGF-β_1_-stimulation such as the fibroplasia model [11, 12], the ‘scar-in-a-jar’ assay combines TGF-β_1_ stimulation with macromolecular crowding to accelerate ECM protein maturation and incorporation into a physiological ECM within a short time-frame. The addition of a neutral hydrophobic polysaccharide such as Ficoll, generates pseudo-3D cell culture conditions described as the excluded volume effect (EVE) [13]. This enhances enzyme activity and induces a rapid cross-linking of deposited collagens and other ECM components [13] enriched with proteins containing the necessary post-translational modifications [11]. This phenotypic in vitro assay permits the identification of novel compound entities that may interfere with collagen transcription, translation and post-translational modification by disrupting type I collagen fibrillogenesis that more closely resembles the mature fibres characteristic of fibrotic lesions found in fibroproliferative diseases. Here we describe the optimisation and implementation of the ‘scar-in-a-jar’ assay using primary IPF patient lung fibroblasts into a robust, medium-throughput, high content screening (HCS) assay for the identification and annotation of novel anti-fibrotic agents.

## Results

### Development of a high content, medium throughput type I collagen deposition assay applying macromolecular crowding in disease-derived fibroblasts

**Fig. 1:**
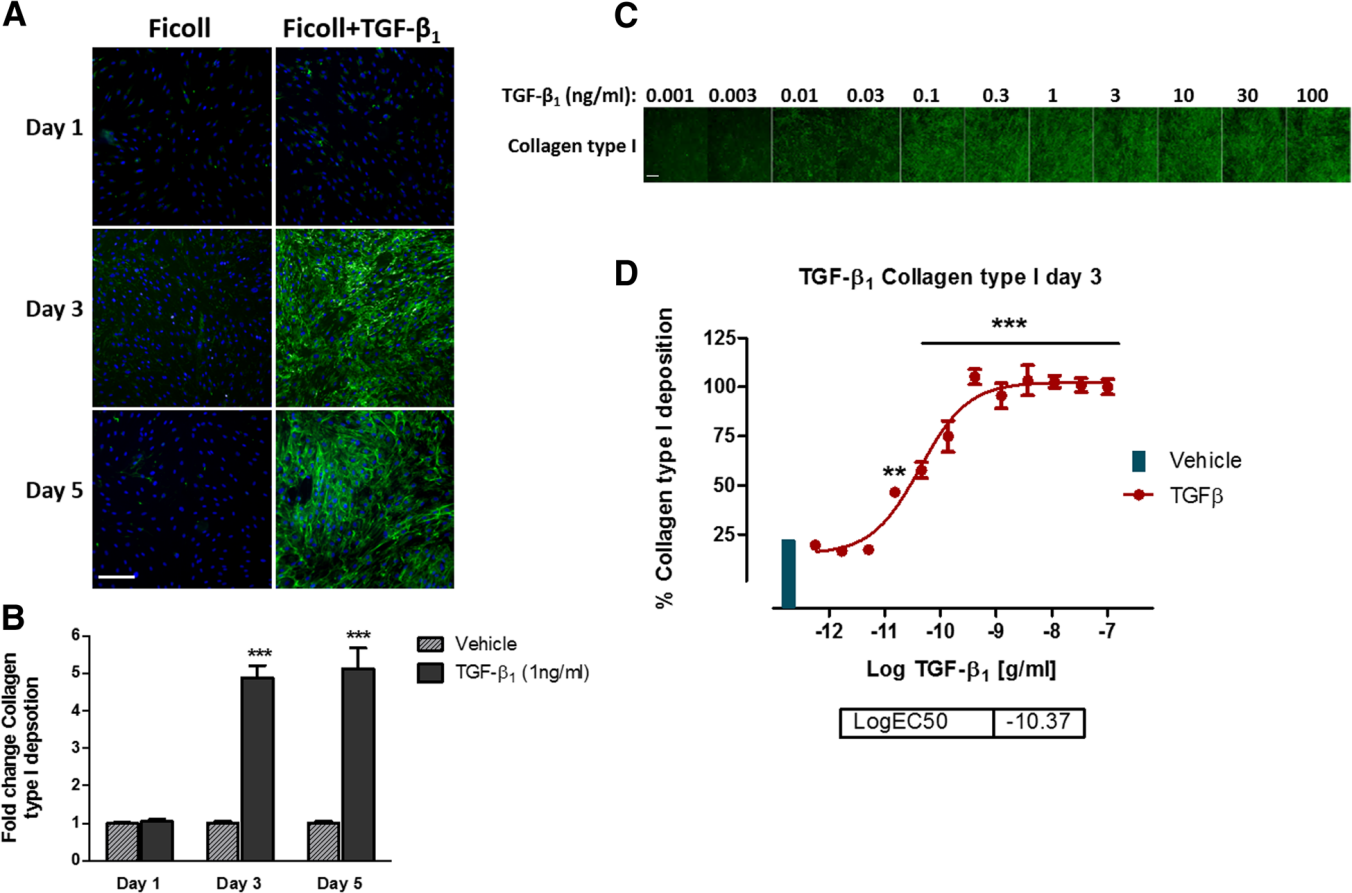
Effects of TGF-β_1_ stimulation in combination with macromolecular crowding on collagen type I deposition in vitro*.* **a** Confluent IPF lung fibroblasts were incubated with TGF-β_1_ in media containing ficoll (PM70 & PM400) and ascorbic acid. After 1, 3 and 5 days, immunocytochemical analysis of deposited collagen type I (Alexa-488) was assessed (representative images). **b** Mean fluorescent intensity of deposited collagen type I represented in (**a**). **c** Immunocytochemical analysis of the effects of TGF-β_1_ concentration response on collagen type I deposition from IPF fibroblasts after 72 h. **d** Quantification of collagen deposition (**c**), after 72 h TGF-β_1_ stimulation of IPF lung fibroblasts. All data points and images are representative of 3 independent experiments using 10x magnification. Scale bars represent 200 μm. Data points represent mean ± SEM. **P ≤ 0.01, ***P ≤ 0.001 as determined by Mann-Whitney (**b**), or one-way ANOVA (**d**)

Initially we sought to develop the ‘scar-in-a-jar’ assay [11, 14] into a high content, medium throughput screening assay for the rapid identification of novel anti-fibrotic compounds. We assessed the effects of macromolecular crowding conditions (Ficoll media) on collagen type I deposition from IPF lung fibroblasts in the presence and absence of TGF-β_1_ in a time-dependent manner (Fig. 1a and b). Immunocytochemical analysis of deposited type I collagen confirmed that in the absence of TGF-β_1_, macromolecular crowding media has a negligible effect on mature fibrillar type I collagen formation. Similar results were observed after 24 h, following TGF-β_1_ addition (Fig. 1a). However, after 3 days TGF-β_1_ induced approximately a 5-fold increase in collagen type I deposition compared to minimal deposited collagen in response to crowding media alone and this signal:background ratio was maintained for up to 5 days in culture (Fig. 1b). While TGF-β_1_ stimulation in the absence of macromolecular crowding induces collagen production after 3 days, the majority of collagen type I immunoreactivity is intracellular rather than deposited into the ECM in mature fibrils (data not shown).

**Fig. 2:**
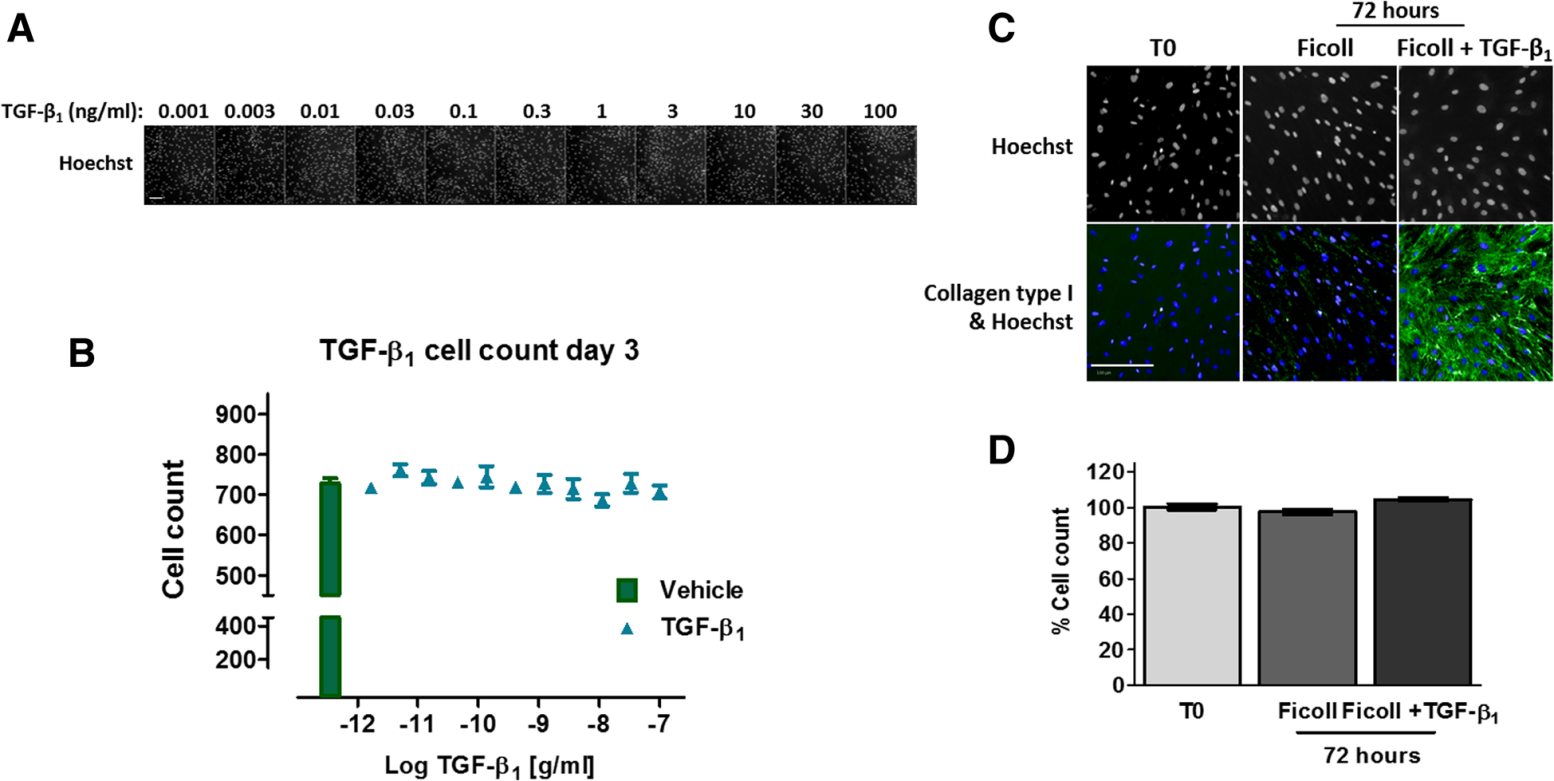
Effects of TGF-β_1_ stimulation in combination with macromolecular crowding on IPF fibroblast nuclear integrity in vitro***.*** **a** Immunocytochemical images of Hoechst stained IPF fibroblast nuclei after exposure to increasing concentrations of TGF-β_1_ for 72 h. **b** Quantification of cell number identified in (A) using Hoechst-stained nuclei and computational algorithm to define nuclei area. **c** & **d** Images and quantification of comparison of cell number at time zero (T_0_), and after 72 h TGF-β_1_ (1 ng/ml) in the presence of Ficoll media. Quantification is represented as % cell count in comparison to the number of cells at the start of the experiment (T_0_). Cell counts were quantified from two fields of view at 10x magnification. Scale bars represent 200 μm. Images are representative of at least 3 independent tests. Data points represent mean ± SEM

To determine the optimal concentration of TGF-β_1_ to induce a significant and robust type I collagen deposition assay signal, cells were exposed to TGF-β_1_ concentrations ranging from 100 ng/ml to 1 pg/ml over 3 days (Fig. 1c and d). After 72 h, TGF-β_1_ stimulation significantly enhanced type I collagen deposition by IPF lung fibroblasts at concentrations of 15 pg/ml and above, reaching effective concentration (EC)_80_ at 0.5 ng/ml. TGF-β_1_ can exert both pro and anti-proliferative effects and has previously been shown to induce fibroblast proliferation at low concentrations in the range of 5 pg/ml [15]. To ensure that the increases in collagen deposition were not dependent on cell number, we assessed the effects of TGF-β_1_ stimulation on total cell count. Cell number per FOV were calculated based on the detection of Hoechst-stained nuclei and compared to vehicle-treated cells (Fig. 2a and b). Furthermore, the total cell count at the time of TGF-β_1_ addition (T_0_) was compared with cell counts 3 days after TGF-β_1_ addition (Fig. 2c and d). Overall, these data suggest that TGF-β_1_-induced collagen deposition in the ‘scar-in-a-jar’ assay is independent of cell number under these culture conditions. Furthermore, TGF-β_1_ has a negligible effect on IPF lung fibroblast cell growth over 72 h when cells were grown to confluence.

**Fig. 3:**
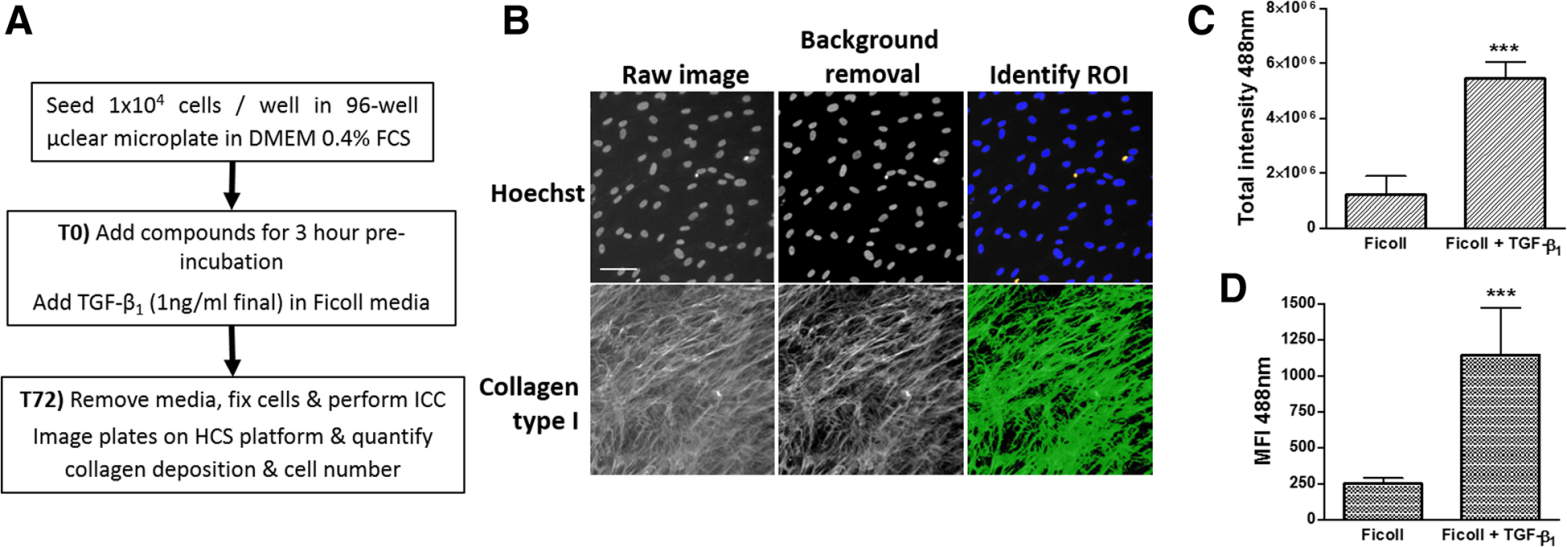
Overview of the ‘scar-in-a-jar’ assay and HCS algorithm to analyse nuclear integrity and collagen deposition*.* **a** Overview of the ‘scar-in-a-jar’ assay from seeding cells on the first day to adding compounds, TGF-β_1_ stimulation on the second day (T_0_), cell fixation and performing immunocytochemistry (ICC) to quantify cell number and collagen deposition at 72 h post-stimulation (T_72_). **b** Representative images of Hoechst-stained nuclei (350 nm/460 nm) and AlexaFluor488 (490 nm/525 nm)-collagen type I immunoreactivity acquired using the CellInsight at 10x magnification. A computational algorithm identified viable Hoechst-stained nuclei (blue) and excluded nuclei (yellow). Green indicates collagen mask applied to fluorescence above the background signal. Scale bars represent 200 μm. **c** & **d** Using a modified version of the ‘Cell Health Profiling v4’ algorithm, total fluorescent intensity (**c**) and mean fluorescent intensity (MFI; (**d**)) were quantified. Data points represent mean ± SEM (*n* = 9 independent experiments). ***P ≤ 0.001 as determined by Mann-Whitney statistical analysis

High content screening and image analysis requires a computational algorithm to accurately and robustly quantify parameters from large numbers of images at the subcellular level in multiple cells. Therefore, it is important to develop an algorithm that is versatile and requires minimal adjustment between assays regardless of inter- and intra-plate variation and biological variability [16]. A computational algorithm was optimized to quantify ECM-associated-type I collagen as well as accurately calculate the number of viable nuclei per treatment group. Using the ‘Cell Health Profiling v4’ algorithm, the raw images from both channels (350 nm/460 nm and 490 nm/525 nm) were normalised to exclude non-specific, background fluorescence thus reducing inter-well variability. The primary objects (nuclei) were then identified in channel 1 (Hoechst) based on fluorescent intensity above the background signal. Artefacts and cells undergoing apoptosis were also identified and excluded based on variations in size, morphology and fluorescence intensity (Fig. 3b). Following identification of Hoechst-stained nuclei, the algorithm applied a circular mask to surround each nuclei and this was further expanded to define the extracellular area and designate a region of interest (ROI) for collagen deposition (Alexa488). Alexa488 immunofluorescent signal above non-specific background signal within the ROI was considered to be deposited type I collagen. Parameters such as collagen coverage area, total fluorescent intensity and mean fluorescent intensity (MFI) were also calculated for each image (Fig. 3c and d). The collagen area coverage was markedly increased in cells exposed to TGF-β_1_ compared with vehicle-treated cells when a fluorescence intensity threshold was applied (data not shown), however this increased the inter-assay variability for other parameters, therefore a signal threshold was not applied for routine high content screening. Similarly, TGF-β_1_ stimulation induced a significant increase in both the total fluorescent intensity and mean fluorescent intensity (MFI) for collagen deposition compared with vehicle treated-wells (Fig. 3c and d). Overall, MFI represented a more reliable and robust image analysis parameter compared with total intensity, exhibiting a larger assay signal:background ratio and robust Z’ factors (data not shown).

### ‘Scar-in-a-jar’ high content screening assay validation

**Fig. 4:**
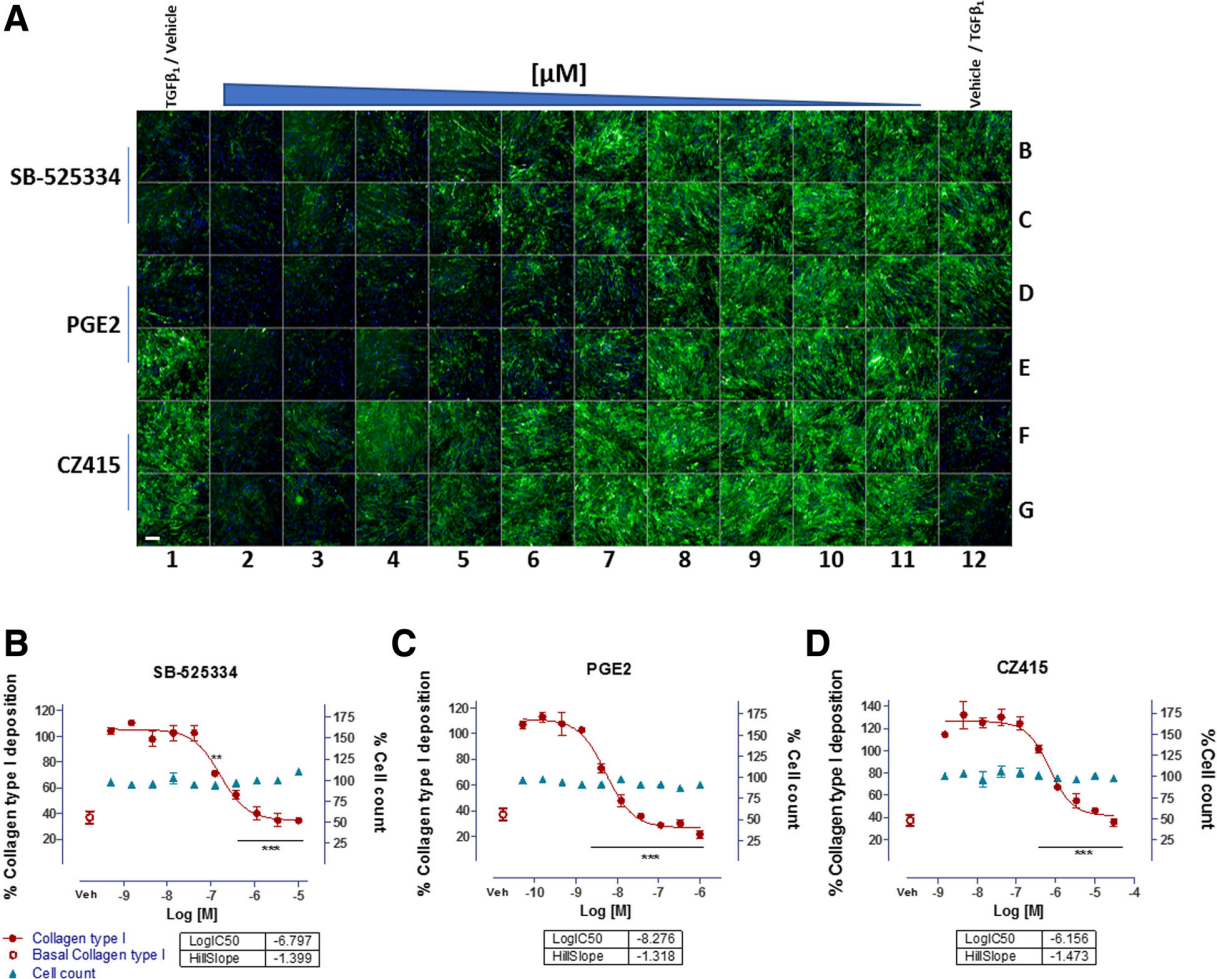
‘Scar-in-a-jar’ high content screening: compound potency (pIC_50_) determination. **a** Representative immunocytochemical image of 96-well plate after 72 h ‘scar-in-a-jar’ assay. Wells B1, C1, D1, E12, F12, G12 were treated with 0.1% DMSO (vehicle) in ficoll media, and wells E1, F1, G1, B12, C12, D12 were treated with vehicle in the presence of TGFβ_1_ [1 ng/ml] in ficoll media. Positive controls, SB-525334 (Alk5 inhibitor), PGE_2_ and CZ415 (mTOR inhibitor) were assayed in a 10-point concentration response in duplicate (0.1% DMSO). Rows A and H were filled with PBS to minimise plate effect. Wells were stained for collagen type I, and Hoechst to visualise ECM deposition and cell count. Images were acquired using a CellInsight HCS microscope at 10x magnification. Scale bars represent 200 μm. **b**-**d** Representative screening data from assay positive controls (**b**) SB-525334 (Alk5 inhibitor), (**c**) PGE_2_ and (**d**) CZ415 (mTOR inhibitor). Graphs indicate the quantification of collagen type I deposition (Alexa488) and cell count (nuclei count, Hoechst) as determined by immunocytochemical analysis of IPF lung fibroblasts after 72 h. Vehicle (Veh) data points represent basal collagen deposition in the presence of 0.1% DMSO. Data points were plotted from mean ± SEM from 10-point concentration response curves in duplicate (**a**). **P ≤ 0.01, ***P ≤ 0.001 one-way ANOVA. 10x magnification

To assess whether the high content ‘scar-in-a-jar’ assay was a reliable, robust screening tool for the identification of novel anti-fibrotic compounds, we assessed the potencies of compounds known to inhibit collagen deposition. Inhibitors of TGF-β_1_ signalling such as ALK5 (SB-525334 [14]), PGE_2_ [17, 18], and mechanistic target of rapamycin (mTOR) inhibitor (CZ415 [19]) were chosen as positive control mechanisms known to interfere with collagen synthesis (Fig. 4).

IPF lung fibroblasts seeded in 96-well plates were exposed to compounds in duplicate for a 3 h pre-incubation at 37 °C prior to stimulation with TGF-β_1_ under crowding conditions for 72 h. Plates were fixed and following immunocytochemistry the MFI for deposited type I collagen was determined and sigmoidal concentration response curves plotted to evaluate potency (pIC_50_) and the Hill slope coefficient. Cell counts were also derived to indicate potential compound cytotoxicity (Fig. 4). To date, the high content ‘scar-in-a-jar’ screening assay has been performed multiple times calculating 480 IC_50_ data points, screening 141 novel anti fibrotic compounds, and achieving over a 95% success rate. The average assay signal to background ratio of 4.6 represents the fold change in collagen deposition between TGF-β_1_-stimulated and vehicle-exposed cells, achieving Z’ values of 0.49–0.51 confirming assay robustness. SB-525334 and CZ415 exhibited average inhibitory potencies of 276 nM (+/− 188 nM *n* = 28) and 421 nM (+/− 323 nM *n* = 25) respectively with low %CV (coefficient of variance) of under 15% (Table 1). In contrast, PGE_2_ exhibited a superior potency of 22 nM (+/− 242 nM *n* = 8) however the %CV and standard deviations were also greater indicating inter-assay variability and potentially a less reliable positive control.

**Table 1:** ‘Scar-in-a-jar’ HCS assay metrics and compound potency for standard inhibitors

	pIC_50_			Assay metrics		
	MEAN	STDEV	%CV	Assay window	n	Z prime
PGE_2_	7.65	0.98	12.7	4.64	8	0.50
SB-525334	6.79	0.33	4.8	4.63	28	0.49
CZ415	6.38	0.31	4.8	4.67	25	0.51

Average pIC_50_, standard deviation (STDEV), and % coefficient of variance (%CV) values of the assay controls: SB-525334, PGE_2_ and CZ415 and assay parameters to measure assay performance and robustness

### The ‘scar-in-a-jar’ assay increases fibroblast activation and ECM deposition in vitro

**Fig. 5:**
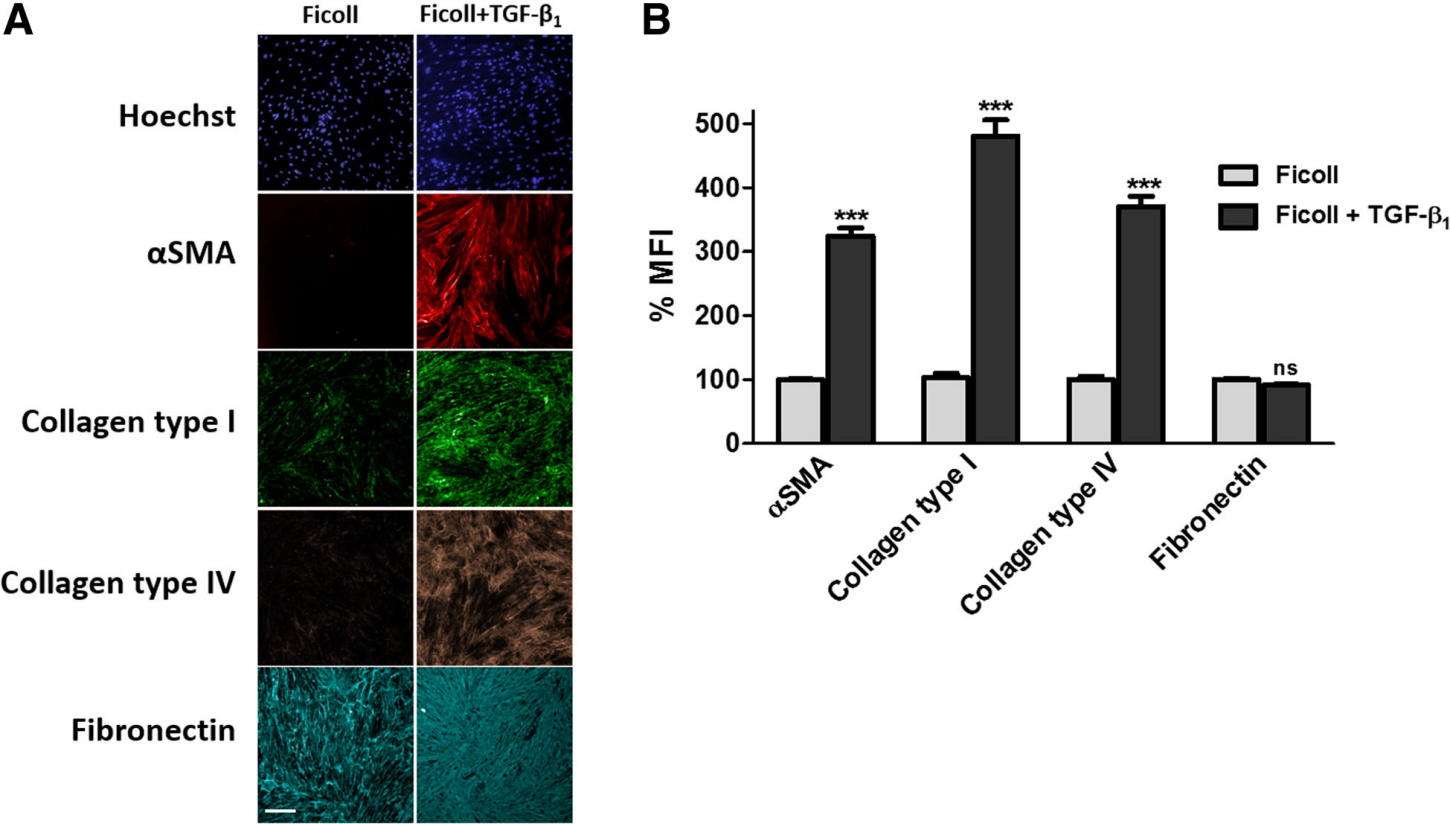
Markers of myofibroblast activation and extracellular matrix deposition. **a** Immunocytochemical analysis of fibroblast activation (α-SMA) and ECM deposition (collagen type I, collagen type IV and fibronectin) in response to TGF-β_1_ stimulation [1 ng/ml] for 72 h. Images are representative of *n* = 3 independent experiments at 10x magnification. Scale bars represent 200 μm. **b** mean fluorescent intensity of markers in (A) expressed as percent mean fluorescent intensity (MFI) normalised to ficoll treated-vehicle control. Histogram represents mean ± SEM. ***P ≤ 0.001 as determined by Mann-Whitney statistical analysis

The activation of fibroblasts to a myofibroblast phenotype is a hallmark feature of tissue remodelling pathologies including fibrosis and cancer (cancer associated fibroblasts) [1, 8]. We sought to determine whether other markers of ECM deposition and fibroblast activation are detectable in the ‘scar-in-a-jar’ assay using HCS (Fig. 5). After 72 h stimulation with TGF-β_1_ under macromolecular crowding conditions, the expression of α-SMA as a marker of fibroblast to myofibroblast activation, and the ECM proteins collagen type IV and fibronectin was assessed. TGF-β_1_ (1 ng/ml) in crowding conditions significantly induced (*P* ≤ 0.001) a 3.2-fold increase in α-SMA, a 4.7-fold increase in collagen type I and a 3.7-fold increase in collagen type IV. Although TGF-β_1_ stimulation induced a redistribution of deposited cellular fibronectin fibres, there was no significant effect on the MFI of fibronectin after 3 days possibly due to high baseline levels of ECM-incorporated fibronectin (Fig. 5b).

## Discussion

Given the lack of reliable, disease relevant in vivo models of fibroproliferative diseases, anti-fibrotic drug discovery relies heavily on phenotypic in vitro models. With the recent advancement of disease relevant 3D cell culture models taking favour over traditional 2D models, there is a clear need for the development of reliable inflammation-independent in vitro models of fibrosis and tissue remodelling [20, 21]. Here we have described a phenotypic in vitro collagen deposition assay enabling accurate quantification of rapidly deposited, insoluble, cross-linked collagen that can be combined with patients-derived cells in a medium throughput manner. The ‘scar-in-a-jar’ assay has been previously described [11, 13] and utilised to confirm efficacy of novel anti-fibrotic agents [2, 10, 14], however until now a high content, medium throughput screening assay has not been described. The ability to screen compounds using 10-point concentration response curves enables the generation of accurate potency and efficacy data for novel compounds inhibiting collagen deposition at multiple points during processing and maturation rather than focussing on transcriptional read-outs or soluble immature collagen monomers. Furthermore, this assay gives an early indication of potential cytotoxicity, by assessing nuclei size, morphology, and intensity. These parameters identify nuclei blebbing, shrinkage and fragmentation, all of which are indicators of apoptosis [22].

Here we have combined macromolecular crowding with the pro-fibrotic and pleiotropic cytokine TGF-β_1_ known to be elevated in IPF [9], to develop a robust, high content, phenotypic screening assay using patient-derived pulmonary fibroblasts. This assay has been used to screen over 150 novel compounds with an assay success rate of over 95%. The control compounds SB-525334 and CZ415 have proven to be reliable positive controls indicating the importance of the TGF-β_1_ [20] and mTOR [2, 14] pathways in type I collagen fibrillogenesis. However, the biological heterogeneity between IPF patient cell lines was prevalent when evaluating the effects of PGE_2_ on collagen deposition. PGE_2_ exhibited a larger range in efficacy compared to other compounds with higher variability in potency estimation suggesting that PGE_2_ is not a robust assay control. This could be due to some IPF patients exhibiting a deficiency in PGE_2_ synthesis coupled with an inability to respond to exogenously added eicosanoid following downregulation of PGE_2_ receptors and signalling [18, 23–25]. On the one hand, this supports the use of patient-derived cells to more accurately reflect the disease biology, however this also highlights the challenges of using multiple patient primary lines to generate accurate potency data. While subtle differences may be observed at baseline between healthy control and IPF primary lung fibroblasts, these differences become undetectable upon TGF-β_1_ stimulation (data not shown). Recently we demonstrated that healthy control lung fibroblasts also exhibit a similar TGF-β_1_-induced collagen type I response [2].

While type I collagen is considered to be one of the most significantly upregulated ECM proteins, and a hallmark feature of fibroproliferative diseases [8], hits from the ‘scar-in-a-jar’ assay can be further validated against other markers of fibrosis. We demonstrate that in addition to quantifying type I collagen deposition, the ‘scar-in-a-jar’ assay also enables the visualisation and quantification of collagen type IV [26] and fibronectin deposition, as well as α-SMA expression. Indeed this assay could be utilised to quantify a range of ECM proteins, a number of which have previously been reported to upregulated by TGF-β_1_ and macromolecular crowding including collagens I, III, IV, V, VI, XII [27]. Recently it has been demonstrated that culturing fibroblasts on stiff substrates such as tissue culture plastic induces cellular fibronectin production [28], perhaps causing the high basal fibronectin deposition. However while cellular fibronectin deposition was not elevated in the ‘scar-in-a-jar’ assay, there was a marked difference in fibronectin distribution and organisation; a characteristic considered to be an important pathological event in fibrosis [29], thus representing another potential parameter for high content analysis.

The ‘scar-in-a-jar’ assay offers several advantages over previous in vitro models of fibrosis. Unlike previous techniques, the ‘scar-in-a-jar’ assay enables the visualisation and quantification of rapid deposition and cross-linking of mature collagen fibrils, more closely resembling the disorganized fibres characteristic of IPF lesions [11, 13, 30]. In contrast, previous assays have focused on quantifying soluble collagens using the Sircol assay [31] or measuring soluble P1NP (procollagen type I N-terminal propeptide [32]), both of which reflect surrogate markers of ECM turnover [33]. Other methods of quantifying ECM deposition include the histological Picro-Sirius red staining, however this lacks resolution and collagen specificity [34]. Similarly, quantification of hydroxyproline, using reverse-phase HPLC, is non-specific and involves substantial sample processing and manipulation that is both low throughput and time-consuming [11].

The addition of macromolecular crowding agents to culture media has previously been demonstrated to mimic the dense extracellular microenvironment by imitating the features of the tissues from which the cells were isolated [35, 36]. Macromolecular crowding enhances ECM deposition as well as influencing the alignment, thickness, and architecture of ECM fibrils in vitro [11, 30, 35, 36]. Collagen chains which are synthesised in the endoplasmic reticulum, and undergo post translational modifications (PTMs) such as the hydroxylation of lysine and proline residues, followed by glycosylation of specific hydroxyl residues [37]. Following PTMs, collagen chains (two proα1 and one proα2) form a triple helix to make pro-collagen which is released into the extracellular space [37]. The N- and C- procollagen terminals are cleaved by ADAMTS and procollagen C-proteinase (bone morphogenic protein-1/BMP1) [30] and cross-linked to form mature collagen fibrils. Macromolecular crowding induces the phenomenon known as the excluded volume effect (EVE) to dramatically enhance enzymatic activity and accelerate ECM formation in vitro. In combination with TGF-β_1_, macromolecular crowding-induced EVE significantly elevates the deposition of mature, cross-linked ECM [11, 13, 30]. To increase the likelihood of identifying novel inhibitors of ECM synthesis and maturation with unknown mechanisms of action, a 3-h pre-incubation step is performed to allow compounds to reach equilibrium before TGF-β_1_ stimulation.

We have described the development of an IPF-relevant ‘scar-in-a-jar’ assay to screen novel anti-fibrotic compounds. Recently, we also utilised the ‘scar-in-a-jar’ assay to identify novel anti-fibrotic targets using CRISPR/Cas9 gene editing to explore the cellular phenotype in response to alterations in genotype [2, 38]. With the recent advances in whole genome CRISPR screening [39], the HCS ‘scar-in-a-jar’ assay represents an attractive approach to identify novel targets. Indeed, this assay is not limited to IPF research. The benefits of macromolecular crowding in tissue-specific cultures have been described for a number of other fibrotic and tissue remodelling assays [40] including using corneal fibroblasts [36], dermal fibroblasts [41] and bone marrow stroma-derived cells [42] affecting ECM deposition as well as modifying the cellular phenotype [43]. Furthermore, this assay could be developed to explore the impact of ECM deposition in complex multi-cellular systems. For example, others have utilised macromolecular crowding-induced ECM to explore the effects of different tissue microenvironments on cell-ECM interactions including mesenchymal [43] and embryonic [27] stem cells. Recently, cancer-associated fibroblast (CAF)-derived ECM has been shown to be deposited in an organised, aligned manner facilitating cancer cell motility and enhancing tissue invasion [44–46]. In addition to its application for pulmonary fibrosis research as outlined in this study, the HCS ‘scar-in-a-jar’ assay could be utilised to study complex cell-cell and cell-ECM interactions in research areas including fibrosis (pulmonary, hepatic, renal, cardiac and dermal), tissue remodelling, wound repair and oncology.

## Conclusion

In conclusion, the HCS ‘scar-in-a-jar’ model represents a robust, disease-specific in vitro model of IPF ECM turnover, in a high-content, medium-throughput format capable of screening large numbers of novel compounds for anti-fibrotic drug discovery. This assay could potentially facilitate further studies to explore cell-matrix interactions in complex multi-cellular systems and identify new targets for novel therapies.

## Methods

### Ethical approval

Primary human lung fibroblasts were obtained from IPF patients undergoing lung transplant therapy. All patients gave full consent and all procedures were performed in line with research ethics committee approval (11/NE/0291). Human biological samples were sourced ethically and their research use was in accord with the terms of the informed consents under an IRB/EC approved protocol. All experiments were performed in accordance with relevant guidelines and regulations.

### Cell culture

Human lung fibroblasts were derived by explant culture from IPF patient lung tissue as previously described [15]. Under sterile conditions, biopsies were washed and finely cut into 1mm^3^ fragments before adhering to 10 mm petri dishes and culturing in Dulbecco’s Modified Eagle Medium (DMEM) (Gibco, 21,969) supplemented with 4 mM L-glutamine and 10% heat inactivated (HI) FCS (Gibco, 10,270,106), 100 U/ml penicillin, 100 μg/ml streptomycin (Invitrogen, 15,140–122) and amphotericin B (0.25 μg/ml). Primary cells were grown in DMEM 10% HI FCS and 4 mM L-glutamine at 37 °C, 10% CO_2_, in a humidified environment and routinely grown to approximately 90% confluence before passaging. After undergoing several population doublings, mycoplasma clear cultures (as defined by ATCC Universal Mycoplasma Detection Protocol, Cat# 30-1012 K) were cryopreserved in liquid nitrogen. Experiments were performed on cells between passages 4 to 8.

### ‘Scar-in-a-jar’ in vitro high content screening model for fibrosis

IPF fibroblasts were trypsinised (Gibco, 25,300,062) and seeded into black-walled 96-well imaging plates (BD Falcon, 353,219) at a density of 1 × 10^4^ cells per well in DMEM supplemented with 4 mM L-Glutamine and 0.4% FCS (DMEM 0.4%). Fibroblasts were left for 24 h at 37 °C, 10% CO_2_ to reach confluence in assay plates. Once confluent, TGF-β_1_ (1 ng/ml final, unless otherwise stated, R&D Systems, 100-B-01) was added to fibroblasts in ‘Ficoll’ media; containing L-Ascorbic acid (50 μg/ml final, Sigma, A8960) and the hydrophobic polysaccharides Ficoll-PM70 (37.5 mg/ml final, Sigma, F2878) and Ficoll-PM400 (25 mg/ml final, Sigma, F4375) in DMEM 0.4%.

For compound screening, fibroblasts were incubated with either vehicle (DMSO at 0.1% v/v) or inhibitor (0.1% DMSO final) for a 3 h pre-incubation, before the addition of TGF-β_1_ in Ficoll media and culture at 37 °C, 10% CO_2_. Compound screens were performed over 72 h unless otherwise stated. Concentration responses of PGE_2_ (Prostaglandin E_2_; Sigma, P0409), SB-525334 (activin-like kinase receptor (ALK)5 inhibitor; Sigma, S8822) and CZ415 (mammalian target of rapamycin; mTOR inhibitor; [19]) were tested in every assay as positive controls. Potencies of these control compounds were used to assess assay performance and robustness.

### Immunocytochemistry

To image and quantify the deposition of ECM proteins, confluent monolayers of fibroblasts were fixed in ice-cold methanol for 2 min before washing in PBS with a 96-well platewasher (BioTek 405 TS). Cells were incubated with primary antibodies against α-SMA (Sigma, C6198), collagen type I (Sigma, C2456), collagen type IV (eBioscience, 50–9871-82) or cellular fibronectin (eBioscience, 53–9869-82) at 1:1000 in PBS for 1.5 h at RT, or overnight at 4 °C. After washing in PBS-Tween (0.05% v/v), cells were incubated with the appropriate secondary antibodies (AlexaFluor488 or AlexaFluor555; Invitrogen, A11001 and A32732) at 1:500 and Hoechst (Invitrogen, H3570) at 1:10,000 in PBS for 1 h at RT, protected from light. Cells were washed for a final time in PBS-Tween followed by the addition of PBS before acquiring images on the CellInsight High Content Screening platform (ThermoScientific). Two fields of view (FOV) were acquired per well at 10x magnification. Images were quantified using the ‘Cell Health Profiling v4’ algorithm, part of the Cellomics HCS Studio analysis software (version 6.6.0) to determine the cell count and mean fluorescent intensity (MEAN_TargetAvgIntenCh2) per well.

### Statistical analysis

Graphs and concentration response curves were constructed in GraphPad Prism (v5.0.4). Four-parameter, non-linear regression curves were used to calculate pIC_50_ values and Hill Slope coefficients. To measure the robustness of the type I collagen deposition assay window within each plate the Z prime was calculated from the mean (*μ*) deposition from the positive (*p*) and negative (*n*) controls and standard deviation (*σ*) using the following equation:

$$ 1-\frac{3\left(\sigma p+\sigma n\right)}{\mu \rho -\mu n} $$

To quantify inter-plate variability of control compound potency, the % co-efficient of variation (%CV) was calculated using the mean pIC_50_ (*μ*) and the pIC_50_ standard deviation (*σ*) for control compounds, using the equation:

$$ CV=\frac{\sigma }{u}\ x\ 100 $$

Unless otherwise stated, statistical significance was calculated using Mann-Whitney or one-way ANOVA with Dunnett’s multiple-comparison test. Experiments were performed on three different cell lines and technical replicates are denoted by ‘*n*’.

## Data Availability

Data sharing not applicable to this article as no large datasets were generated or analysed during the current study. Please contact corresponding author for specific data requests.

## Abbreviations

ALK5: Activin-like kinase receptor 5
ANOVA: Analysis of variance
CAF: Cancer associated fibroblast
CKD: Chronic kidney disease
CV: Coefficient of variance
DMEM: Dulbecco’s Modified Eagle Medium
ECM: Extracellular matrix
EVE: Excluded volume effect
FOV: Field of view
HCI: High content imaging
HCS: High content screening
HRCT: High-resolution computed tomography
ILD: Intestinal lung disease
IPF: Idiopathic pulmonary fibrosis
MFI: Mean fluorescent intensity
mTOR: Mammalian target of Rapamycin
PGE2: Prostaglandin E2
STDEV: Standard deviation
TGFβ1: Transforming growth factor β1
αSMA: α Smooth muscle actin

## References

[1] Bonnans C, Chou J, Werb Z (2014). Remodelling the extracellular matrix in development and disease. Nat Rev Mol Cell Biol.

[2] Woodcock HV (2019). The mTORC1/4E-BP1 axis represents a critical signaling node during fibrogenesis. Nat Commun.

[3] Scotton CJ, Chambers RC (2007). Molecular targets in pulmonary fibrosis: the myofibroblast in focus. Chest.

[4] Sgalla G (2018). Nintedanib for the treatment of idiopathic pulmonary fibrosis AU - Varone, Francesco. Expert Opin Pharmacother.

[5] Snell N (2016). P272 epidemiology of idiopathic pulmonary fibrosis in the Uk: findings from the british lung foundation’s ‘respiratory health of the nation’ project. Thorax.

[6] Strongman H, Kausar I, Maher TMJAiT (2018). Incidence, Prevalence, and Survival of Patients with Idiopathic Pulmonary Fibrosis in the UK. Adv Ther.

[7] Phan SH (2008). Biology of fibroblasts and myofibroblasts. Proc Am Thorac Soc.

[8] Burgess JK (2016). The extracellular matrix - the under-recognized element in lung disease?. J Pathol.

[9] Fernandez IE, Eickelberg O (2012). The impact of TGF-beta on lung fibrosis: from targeting to biomarkers. Proc Am Thorac Soc.

[10] Herrmann FE (2017). Olodaterol shows anti-fibrotic efficacy in in vitro and in vivo models of pulmonary fibrosis. Br J Pharmacol.

[11] Chen CZC (2009). The scar-in-a-jar: studying potential antifibrotic compounds from the epigenetic to extracellular level in a single well. Br J Pharmacol.

[12] Clark RA (1997). TGF-beta 1 stimulates cultured human fibroblasts to proliferate and produce tissue-like fibroplasia: a fibronectin matrix-dependent event. J Cell Physiol.

[13] Chen C (2011). Applying macromolecular crowding to enhance extracellular matrix deposition and its remodeling in vitro for tissue engineering and cell-based therapies. Adv Drug Deliv Rev.

[14] Mercer PF (2016). Exploration of a potent PI3 kinase/mTOR inhibitor as a novel anti-fibrotic agent in IPF. Thorax.

[15] Keerthisingam CB (2001). Cyclooxygenase-2 deficiency results in a loss of the anti-proliferative response to transforming growth factor-beta in human fibrotic lung fibroblasts and promotes bleomycin-induced pulmonary fibrosis in mice. Am J Pathol.

[16] Ghosh RN, Lapets O, Haskins JR, Taylor DL, Haskins JR, Giuliano KA (2006). Characteristics and value of directed algorithms in high content screening. High Content Screening: A Powerful Approach to Systems Cell Biology and Drug Discovery.

[17] Zhao J (2016). Prostaglandin E2 inhibits collagen synthesis in dermal fibroblasts and prevents hypertrophic scar formation in vivo. Exp Dermatol.

[18] Bozyk PD, Moore BB (2011). Prostaglandin E2 and the pathogenesis of pulmonary fibrosis. Am J Respir Cell Mol Biol.

[19] Cansfield AD (2016). CZ415, a highly selective mTOR inhibitor showing in vivo efficacy in a collagen induced arthritis model. ACS Med Chem Lett.

[20] Xu Q (2007). In vitro models of TGF-β-induced fibrosis suitable for high-throughput screening of antifibrotic agents. Am J Physiol Renal Physiol.

[21] Sundarakrishnan A (2018). Engineered cell and tissue models of pulmonary fibrosis. Adv Drug Deliv Rev.

[22] Elmore S (2007). Apoptosis: a review of programmed cell death. Toxicol Pathol.

[23] Borok Z (1991). Augmentation of functional prostaglandin E levels on the respiratory epithelial surface by aerosol administration of prostaglandin E. Am Rev Respir Dis.

[24] Vancheri C (2000). Different expression of TNF-alpha receptors and prostaglandin E (2) production in normal and fibrotic lung fibroblasts: potential implications for the evolution of the inflammatory process. Am J Respir Cell Mol Biol.

[25] Wilborn J (1995). Cultured lung fibroblasts isolated from patients with idiopathic pulmonary fibrosis have a diminished capacity to synthesize prostaglandin E2 and to express cyclooxygenase-2. J Clin Invest.

[26] Urushiyama H (2015). Role of α1 and α2 chains of type IV collagen in early fibrotic lesions of idiopathic interstitial pneumonias and migration of lung fibroblasts. Lab Investig.

[27] Peng Y (2012). Human fibroblast matrices bio-assembled under macromolecular crowding support stable propagation of human embryonic stem cells. J Tissue Eng Regen Med.

[28] Klingberg F (2018). The fibronectin ED-A domain enhances recruitment of latent TGF-β-binding protein-1 to the fibroblast matrix. J Cell Sci.

[29] Holdsworth G (2017). Quantitative and organisational changes in mature extracellular matrix revealed through high-content imaging of total protein fluorescently stained in situ. Sci Rep.

[30] Lareu RR (2007). Collagen matrix deposition is dramatically enhanced in vitro when crowded with charged macromolecules: the biological relevance of the excluded volume effect. FEBS Lett.

[31] Mingyuan X (2017). Hypoxia-inducible factor-1α activates transforming growth factor-β1/Smad signaling and increases collagen deposition in dermal fibroblasts. Oncotarget.

[32] Veidal SS (2010). Procollagen type I N-terminal propeptide (PINP) is a marker for fibrogenesis in bile duct ligation-induced fibrosis in rats. Fibrogenesis Tissue Repair.

[33] Fish PV (2007). Potent and selective Nonpeptidic inhibitors of procollagen C-proteinase. J Med Chem.

[34] Drifka CR (2016). Comparison of Picrosirius red staining with second harmonic generation imaging for the quantification of clinically relevant collagen Fiber features in histopathology samples. J Histochem Cytochem : official journal of the Histochemistry Society.

[35] Magno V (2017). Macromolecular crowding for tailoring tissue-derived fibrillated matrices. Acta Biomater.

[36] Kumar P (2015). Macromolecularly crowded in vitro microenvironments accelerate the production of extracellular matrix-rich supramolecular assemblies. Sci Rep.

[37] Yamauchi M, Sricholpech M (2012). Lysine post-translational modifications of collagen. Essays Biochem.

[38] Matteo M (2019). Single-Step, High-Efficiency CRISPR-Cas9 Genome Editing in Primary Human Disease-Derived Fibroblasts. CRISPR J.

[39] Hart T (2017). Evaluation and Design of Genome-Wide CRISPR/SpCas9 knockout screens. G3 (Bethesda, Md).

[40] Benny P, Raghunath M (2017). Making microenvironments: a look into incorporating macromolecular crowding into in vitro experiments, to generate biomimetic microenvironments which are capable of directing cell function for tissue engineering applications. J Tissue Eng.

[41] Benny P (2015). Making more matrix: enhancing the deposition of dermal-epidermal junction components in vitro and accelerating organotypic skin culture development, using macromolecular crowding. Tissue Eng Part A.

[42] Prewitz MC (2015). Extracellular matrix deposition of bone marrow stroma enhanced by macromolecular crowding. Biomaterials.

[43] Zeiger AS (2012). Macromolecular crowding directs extracellular matrix organization and mesenchymal stem cell behavior. PLoS One.

[44] Carey SP (2016). Local extracellular matrix alignment directs cellular protrusion dynamics and migration through Rac1 and FAK. Integr Biol : quantitative biosciences from nano to macro.

[45] Hofschroer V (2017). Extracellular protonation modulates cell-cell interaction mechanics and tissue invasion in human melanoma cells. Sci Rep.

[46] Erdogan B (2017). Cancer-associated fibroblasts promote directional cancer cell migration by aligning fibronectin. J Cell Biol.

